# Using Standard Optical Flow Cytometry for Synchronizing Proliferating Cells in the G1 Phase

**DOI:** 10.1371/journal.pone.0083935

**Published:** 2013-12-31

**Authors:** Manuela Vecsler, Itay Lazar, Amit Tzur

**Affiliations:** 1 The Mina and Everard Goodman Faculty of Life Sciences, Bar-Ilan University, Ramat-Gan, Israel; 2 Advanced Materials and Nanotechnology Institute, Bar-Ilan University, Ramat-Gan, Israel; Texas A&M University, United States of America

## Abstract

Cell cycle research greatly relies on synchronization of proliferating cells. However, effective synchronization of mammalian cells is commonly achieved by long exposure to one or more cell cycle blocking agents. These chemicals are, by definition, hazardous (some more than others), pose uneven cell cycle arrest, thus introducing unwanted variables. The challenge of synchronizing proliferating cells in G1 is even greater; this process typically involves the release of drug-arrested cells into the cycle that follows, a heterogeneous process that can truly limit synchronization. Moreover, drug-based synchronization decouples the cell cycle from cell growth in ways that are understudied and intolerable for those who investigate the relationship between these two processes. In this study we showed that cell size, as approximated by a single light-scatter parameter available in all standard sorters, can be used for synchronizing proliferating mammalian cells in G1 with minimal or no risk to either the cell cycle or cell growth. The power and selectivity of our method are demonstrated for human HEK293 cells that, despite their many advantages, are suboptimal for synchronization, let alone in G1. Our approach is readily available, simple, fast, and inexpensive; it is independent of any drugs or dyes, and nonhazardous. These properties are relevant for the study of the mammalian cell cycle, specifically in the context of G1 and cell growth.

## Introduction

The synchronization of proliferating cells offers a strategy to study structural, physiological, and molecular events with respect to the cell cycle – one of the most basic and well-studied processes in biology. For over half a century, methodologies for cell synchronization in prokaryotes, protozoan and metazoan systems have been instrumental in cell-cycle research in the context of normal and malignant proliferation, with clear relevance to cancer and other human diseases.

Cell synchronization in mammalian systems relies, for the most part, on drugs that block the cell cycle and, thus, by definition, are hazardous. Effective synchronization of the average mammalian cell cycle requires single or successive incubations with blocking agents for many hours. Long and uneven cell cycle arrest unavoidably introduces unwanted variables. More specifically, cell cycle blockers decouple the cell cycle from cell growth in ways that are hard to predict and completely understudied [1]. This is an intolerable limitation especially for the study of the cell cycle with respect to cell size and cell growth [2].

Chemical-based synchronization typically blocks the cell cycle in either the M phase, through the activation of the mitotic checkpoint (e.g., taxol, nocodazole), or the S phase, by blocking the DNA replication machinery (thymidine, aphidicolin). More recently, Cdk1 inhibitors (*e.g.,* RO-3306) were introduced as blocking agents of the G2-M transition, despite their high cost [3]. Synchronizing cells in G1 is considerably more challenging because there are no chemicals that truly do so. Thus, G1 populations are normally achieved by releasing cells from drug arrest into the cycle that follows. This is, by definition, suboptimal because both drug release and cell cycle progression are heterogeneous processes to the level that truly limits cell synchronization by the time cells reach G1. These limitations are specific for each cell type; however, overall, they are more profound in cells with a relatively short cell cycle and higher drug sensitivity.

The only established methodology for genuinely synchronizing a large population of proliferating mammalian cells in the G1 phase is the Helmstetter’s ‘baby machine’, which was designed to elute a large amount of newborn cells without any noticeable interference to the cell cycle or cell growth [2], [4]. However, this device is difficult to operate, limited to one or two lymphoblastoid cell lines, unavailable commercially, and seems to be operated in only a handful of laboratories worldwide. Modern versions of the baby machine utilize advanced microfluidic technologies (see, for example, Reference [5]). Although promising, such devices are limited to unattached cells, incompatible with large population size, and rely on cutting-edge technology impractical for most laboratories.

**Figure 1:**
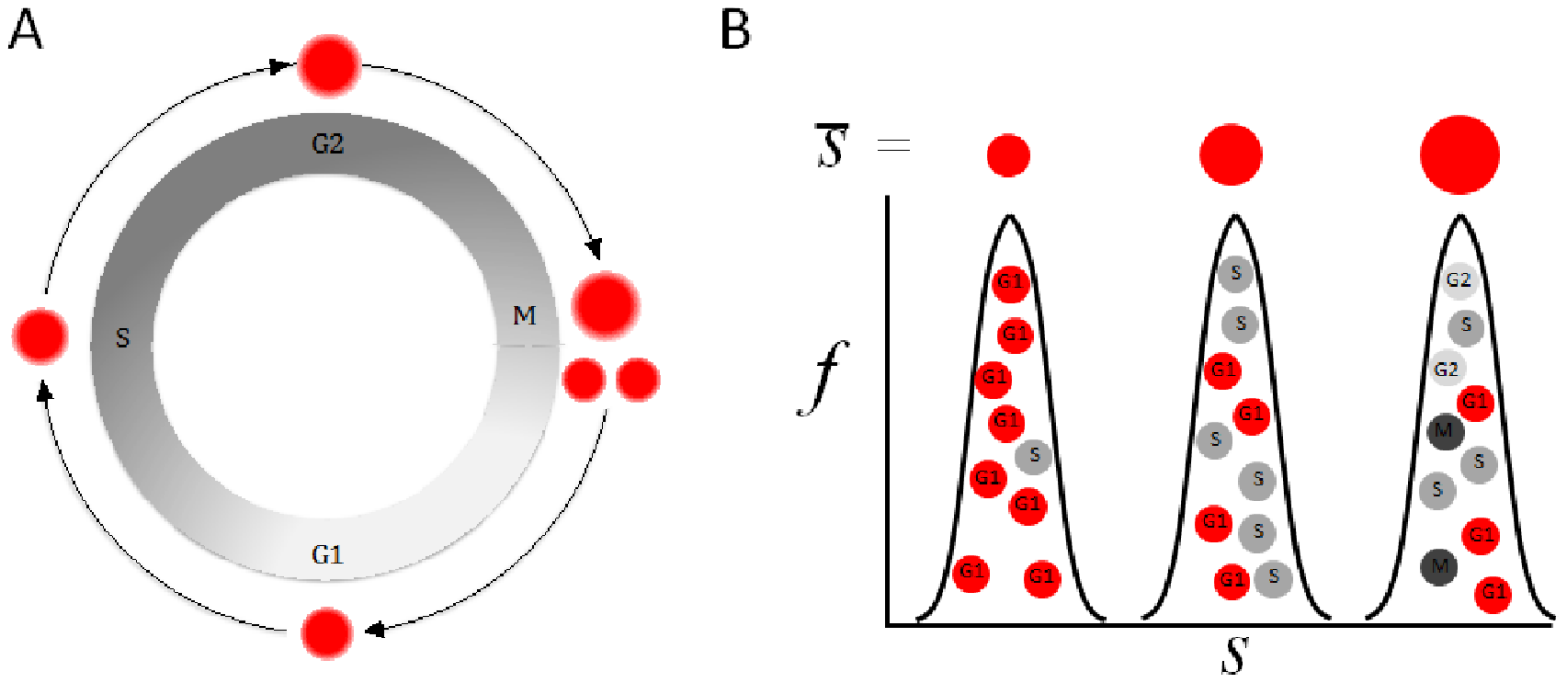
A schematic illustrating the continuous growth of a proliferating cell throughout its life cycle. (A) The overall correlation between geometric size (cell volume) and time from birth enables, in principle, size-based cell cycle synchronization of proliferating cells. (B) Because of the inherent cell-to-cell variability in both, growth rate (cell size over time) and cell cycle progression (also known as the dispersion phenomenon), average cell size, *s*, best correlates with the cell cycle in small rather than large cells (illustrated by the three red circles [top] and the size distributions). The smallest cells are, on the average, the youngest ones (*i.e.,* in G1 phase). The various cell cycle phases are depicted by single letter codes: G1, S, G2, and M.

Cells proliferating in an unchanged environment (steady-state population) maintain a time-invariant cell-size distribution (*i.e.,* the probability density of the cell-size distribution remains constant despite the contentious increase in cell number). We now know, better than before, that cells grow continuously from birth to division [2], [6], [7]. Because of this size-to-‘time from birth’ (*i.e.,* age) correlation, cells of a certain size are likely to be of similar age (see Figure 1). This principle stands behind centrifugal elutriation, which has been long known for its ability to separate uniformly sized cells by gravity. This technique is optimal for purifying budding yeast in G1 by separating young daughter cells from their mothers [8], [9]. Evidently, the method is of limited use in animal cells, perhaps due to its inherent complexity and noticeable unavailability or, alternatively, its preference for round, symmetric, and high-density particles (such as cell-walled organisms) that, most likely, reduce size selectivity in amorphous objects of nearly aqueous density, such as animal cells.

In our previous study, we focused on optimizing standard optical flow cytometry for size (volume)-based separation of mammalian cells [10]. In this report, we demonstrate size-based sorting as a readily available technique for fast, inexpensive, and nonhazardous purification of G1 cells for the purpose of synchronization and, ultimately, for the benefit of the cell cycle research. As a proof of concept, we chose HEK293 cells, an attractive human cell system, most known for its easy transfectability (even with low-cost reagents as calcium phosphate), its biochemical applications, and its simple maintenance, but also for its poor compatibility with cell synchronization, let alone in G1.

## Results and Discussion

### The Light Scatter Parameters FSC-W and SSC-A, but not FSC-A, are Optimal for the Size-based Separation of Proliferating HEK293 Cells

**Figure 2:**
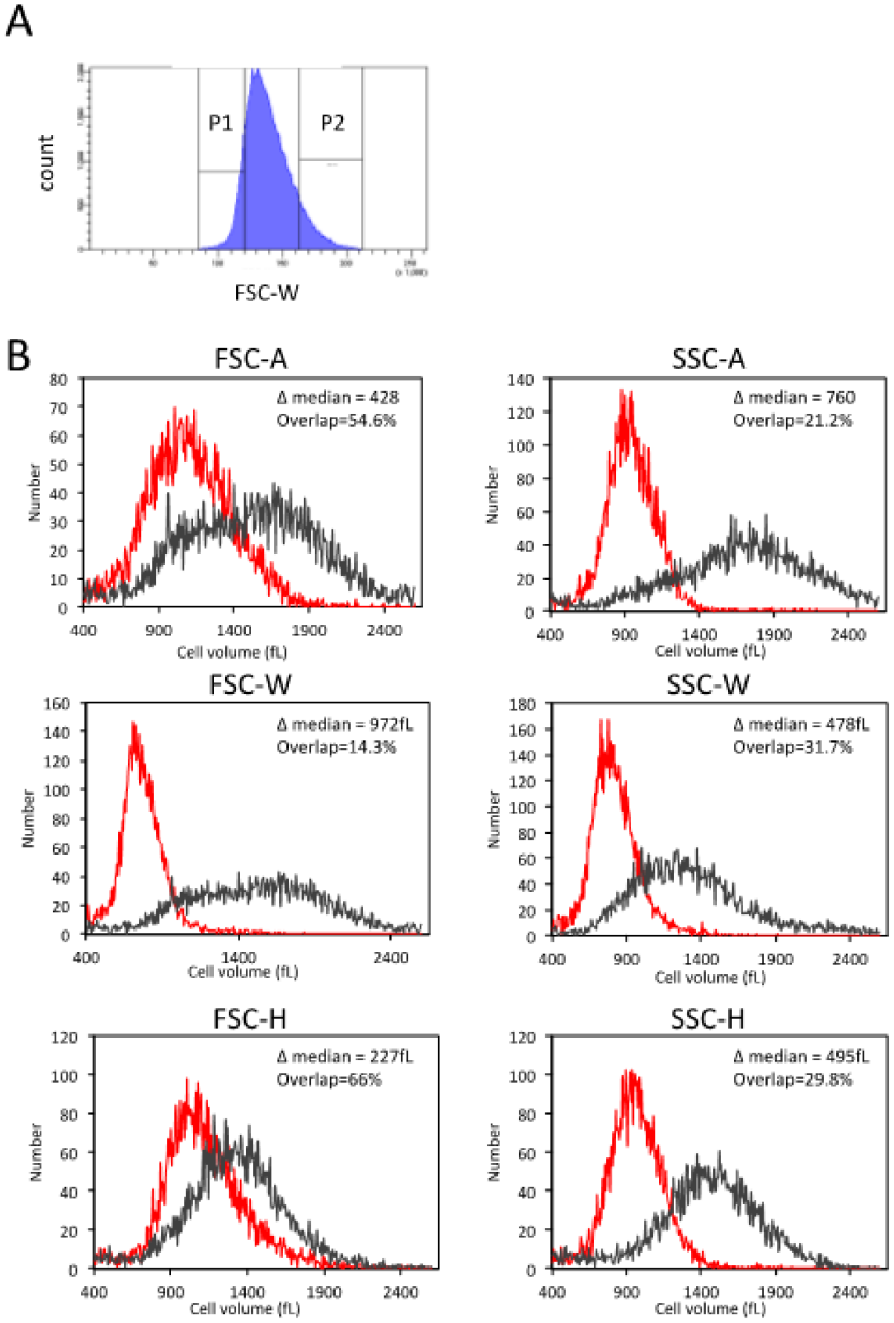
Optimizing size-based sorting of HEK293 cells. (A) The low and high 10% ends of the depicted light-scatter distributions of proliferating HEK293 cells were gated for sorting by FACSAria III. (B) Volume distributions of the sorted low- and high-end cell populations, as determined by Multisizer IV Coulter counter, are plotted in red and black, respectively. We used the calculated percent overlap and the difference in median values (Δ median) between the measured volume distributions of the ‘small’ and ‘large’ sorted cell populations to evaluate the competency of various light-scatter parameters to approximate cell size in HEK293.

We previously showed that the width (W) of the forward scatter pulse (FSC) and the area (A) of the side-scatter pulse (SSC), as measured by standard cytometers, provide a good proxy for cell size, at least in spherical and fairly symmetric cells of hematopoietic origin [10]. In that previous study, we also demonstrated that the optimal light-scatter parameter for size approximation should be determined empirically for each cell type. Because our main goal was to select uniformly sized cells, we first tested which light parameter correlates best with cell size in HEK293. To this end, we used the BD FACSAria III cell sorter and gated the upper and lower 10% of the intensity distribution of the FSC and SSC parameters for sorting (gating strategy is demonstrated for FSC-W in Figure 2A). Volume measurements of sorted cell fractions were subsequently made using a Coulter counter. We used the calculated percentage overlap and the difference in median values (Δ median) between the measured volume distributions of the ‘small’ and ‘large’ sorted fractions to describe the quality of the separation based on various surrogate parameters, and to ultimately determine which of the light scatter parameters best relate to the actual cell size (volume) in HEK293 cells. As shown in Figure 2B, FSC-W and SSC-A signals were found to be the optimal light scatter proxies of cell size for HEK293 cells. FSC-W was slightly advantageous over SSC-A and thus, used as a size surrogate from this point onward. Although subsidiary in the context of this study, these results demonstrate the dominance of FSC-W, as well as SSC-A, over FSC-A in approximating cell size in adherent and amorphous cells, such as HEK293. Hopefully, this observation, supported by our previous study [10], would help remedy the long-lasting misuse of FSC-A in this context.

### Size-based Sorting can Yield a Highly Purified G1 Cell Population

**Figure 3:**
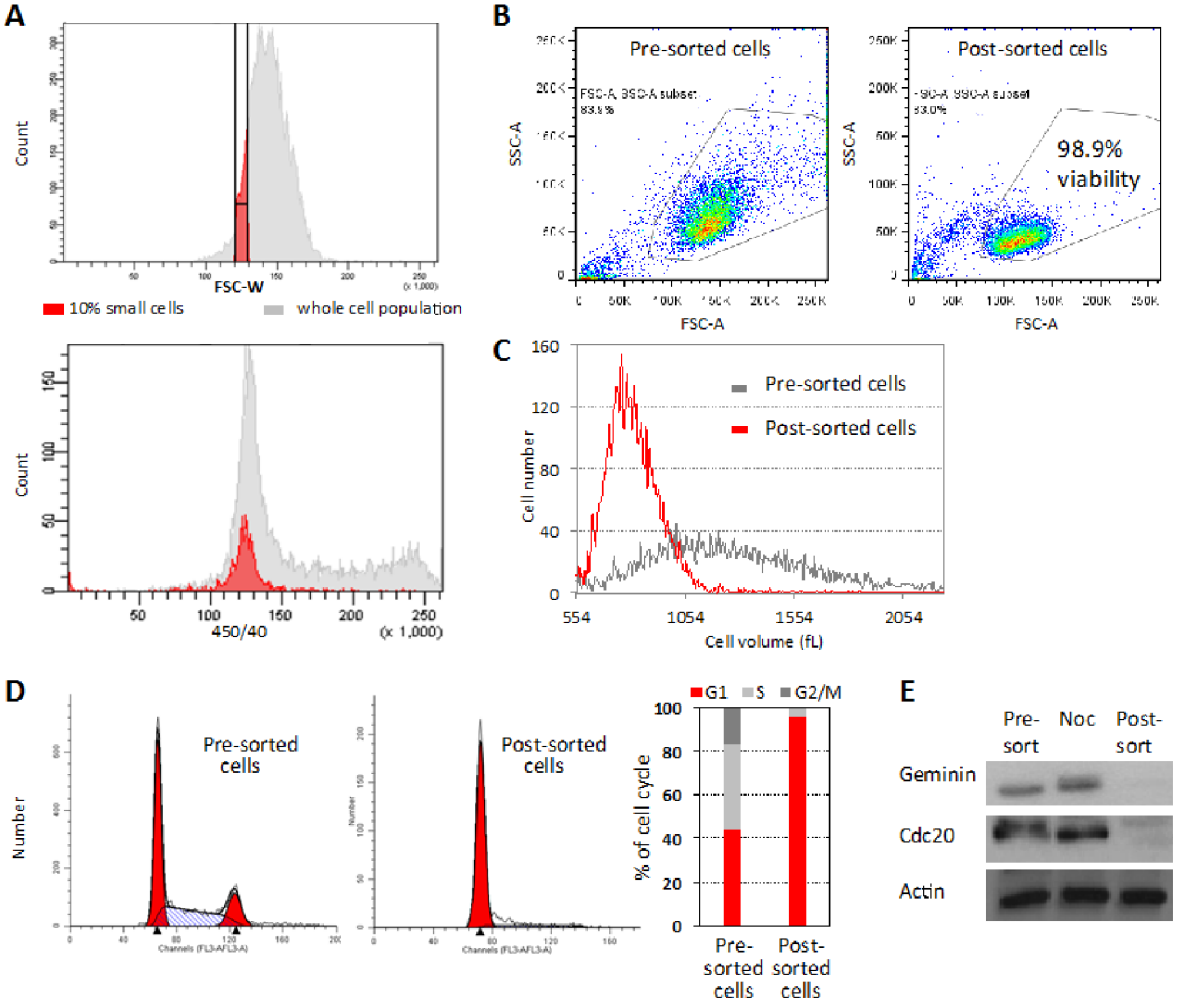
Size-based sorting yields a highly purified G1 cell population. (A) Sorting strategy: FSC-W was used to approximate cell size in an exponentially growing Hoechst-stained HEK293 cell population. Cells exhibiting the lowest 8% FSC-W intensity were gated (marked by the red area at the top panel) and their DNA content was monitored with respect to the total cell population (bottom panel, red *vs.* gray area). Cells at the very left tail of the FSC-W distribution were regarded as outliers and deliberately excluded. (B) Cell viability of post-sorted cells, as estimated by an SSC-A/FSC-A bivariate plot. (C and D) Unstained, normally proliferating HEK293 cells exhibiting the lowest 8% FSC-W intensity were sorted. The post-sort cells were either (C) measured by Multisizer IV Coulter counter to determine cell volume (femtoliter, fL), or (D) fixed and labeled with propidium iodide (PI) for accurately quantifying DNA content and cell cycle distribution (ModFit LT). (E) Protein extracts from nocodazole-arrested, as well as from pre (asynchronous)- and post-sorted (see details in C) HEK293 cells were analyzed by Western blotting to detect the canonical cell cycle proteins Geminin and Cdc20. Actin labeling was used as a loading control. FACSAria III was used for sorting and analyzing live cells, and the Gallios flow cytometer for quantifying DNA by PI staining.

Cells grow continuously throughout their cell cycle. Nevertheless, proliferating cells vary in both, growth rate and cell cycle progression [2], [6]. Thus, average cell size is best correlated with age in small, rather than large, cells. Because the smallest cells are, on the average, the youngest ones, cell size can potentially be used for synchronizing proliferating cells in the G1 phase of the cell cycle (illustrated in Figure 1). Following this principle, we next labeled HEK293 cells with Hoechst 33342 in order to relate FSC-W intensity to the DNA content in living cells. We gated cells showing the lowest 8% FSC-W intensity and quantified their DNA (Figure 3A). It is noteworthy that cells at the very left tail of the FSC-W distribution were excluded from the analysis (Figure 3A) because of our concern that this small subset of cells might possess unusual biophysical properties. Following this protocol, we showed that cells at the low end of the FSC-W distribution are in G1 (see red vs. gray distributions in Figure 3A).

Our ultimate goal was to utilize cytometry for cell synchronization. In order to minimize mechanical perturbations to the sorted cells, we set both the flow rate and pressure to minimum, and used an 85 µm nozzle. We then sorted the cells with the lowest 8% FSC-W signal for viability analysis, volume measurements, DNA quantification, and Western blotting. Following this protocol, cell viability post-sorting reached nearly 99% (Figure 3B), suggesting that HEK293 cells tolerate sorting exceptionally well. Cell viability, as well as size selectivity, could also be appreciated by the volume distributions of the post- *vs*. pre-sorted cells (Figure 3C). Importantly, the DNA distribution of the post-sorted cells, as measured accurately in fixed cells, revealed uniform G1 cell populations (90 to 95% G1 cells post-sorted *vs.* ∼45% pre-sorted) (Figure 3D). Interestingly, both the variance in cell volume (∼20%) and the proportion of G1 post-sorting remarkably matched that of newborn L1210 cells that were eluted from the ‘baby-machine’ [2]. Geminin and Cdc20 are canonical cell-cycle proteins that oscillate throughout the cell cycle. Both proteins are targets of the anaphase-promoting complex/cyclosome (APC/C) E3 Ubiquitin ligase, which mediates protein degradation during mitotic exit and the G1 phase [11]–[14]. The two proteins peak at mitosis, reach their minimum at G1, and start accumulating again at the G1/S transition concomitantly with the APC/C inactivation. We measured the level of Geminin and Cdc20 in i) an asynchronous population; ii) prometaphase-arrested cells (nocodazole); and iii) post-sorted cells. As shown in Figure 3E, both Geminin and Cdc20 peaked at the prometaphase but were barely, if at all, detectable in the post-sorted cells selected by FSC-W-based sizing. In the past, we could not detect such a drastic reduction in any APC/C target in synchronous HEK293 cells following standard ‘block and release’ synchronization protocols (data not shown). Taken together, we concluded that size-based sorting is a simple, fast, accessible, cost-effective, and drug−/dye-free approach for purifying large amounts of G1 cells from a population of proliferating cells.

### Employing Size-based Sorting for Synchronizing Proliferating Cells

**Figure 4:**
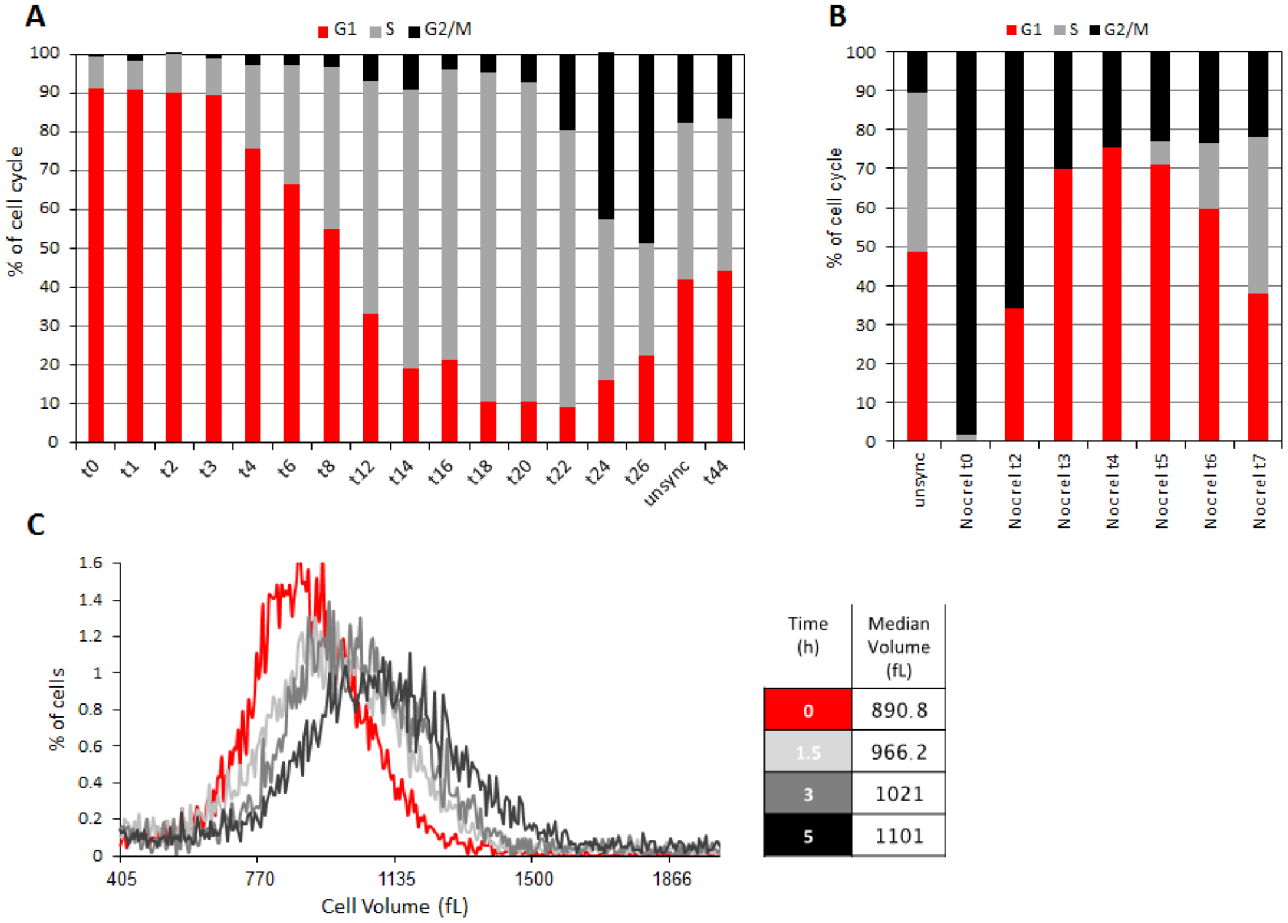
Size-based sorting can be utilized for nonperturbative cell cycle synchronization in the G1 phase. (A) HEK293 cells exhibiting the 8% lowest FSC-W intensity were sorted (FACSAria III) and incubated in fresh, warm media for up to 44 hrs. Cells were harvested at the indicated time points, fixed, and stained with PI for quantifying DNA (Gallios) and cell cycle progression (ModFit LT). DNA quantification of pre-sorted, unsynchronized HEK293 cells (unsync) is also shown. (B) HEK293 cells were synchronized in prometaphase, following the thymidine/nocodazole block. Cells were harvested for PI staining or washed, re-cultured for up to 7 hr, and then harvested. We used the ModFit LT software to assess cell synchronization and cell cycle progression. (C) HEK293 cells were sorted as described in A, and re-cultured immediately in fresh, warm media. Cell volume distribution was measured at 0, 1.5, 3, and 5 hrs post-sorting using the Multisizer IV Coulter counter (see color code on the right). Median values of the four volume distributions are tabled (right).

Although we demonstrated extreme selectivity and viability (Figure 3), post-sorted cells are not guaranteed to progress properly through the cell cycle or to remain synchronous enough for justifying the use of size-based sorting as a *bona fide* ‘synchronization’ method. The reason for this is related to the natural imperfect size-to-age correlation existing in proliferating cells (Figure 1) and the unknown variables that sorting may introduce. Moreover, G1 and S are both long phases. Unlike the S phase, G1 cannot be subcategorized by DNA quantification. Therefore, sorted G1 cells may vary in age in a way that can truly limit synchronization, unless size selectivity can subdivide G1 cells by age. To test that, we followed the sorting strategy described in Figure 3, incubated the sorted cells in fresh warm media, and monitored their cell cycle progression by DNA quantification. As before, the proportion of G1 cells post-sorting exceeded 90%. More importantly, this proportion remained nearly constant for 3 h (see also Figure S1). Cells then started to enter the S phase in a fairly synchronous manner that could be clearly appreciated even 17 h post-sorting, with 85% of the cells in the S phase. Furthermore, we observed a noticeable enrichment of G2/M cells (∼50%) 26 h post-sorting, despite the i) long time distance from sorting (t_0_); ii) natural variability in cell cycle progression (also known as dispersion); and iii) short length of the G2/M phases together (Figure 4A). This level of enrichment is not different from what we observed in the past for L1210 cells that were synchronized as newborns by the ‘baby machine’ [2], and is particularly valuable for those who desire to study mitotic entry and exit without interfering with cytoskeleton dynamics, cell cycle progression, and cell growth. Finally, the cell cycle phase distribution of cells 44 h post-sorting was similar to that of asynchronous cells pre-sorting, evidence of the cells’ health and normality post-synchronization (Figure 4A).

Conceptually, synchronizing cells by size or size-related parameters is, by definition, a compromise because of the inherent imperfect size-to-age correlation in proliferating cells (Figure 1). Yet, the unchanged proportion of G1 cells between 0 to 3 h post-sorting demonstrates the ability to distinguish young from older G1 cells. This intra-G1 cell separation indicates high-resolution synchronization. Alternatively, a delay in S-phase entry could, in principle, result from the biomechanical perturbation inherently affiliated with sorting. As shown in Figure 4C, sizing cells post-sorting indicates immediate and continuous cell growth during G1 and beyond, thus disproving this conjecture. Both cell health (as evident by cell debris; data not shown) and the synchronization quality obtained by our approach were undoubtedly superior to what we could obtain by releasing HEK293 cells from nocodazole block (Figure 4B). In fact, we were unable to get a decent G1-phase population in spite of the close time proximity between the M- and G1- phases, yet further evidence of the heterogeneous release from the nocodazole block [15]. Altogether, the results in Figure 4 not only demonstrate the selectivity of our approach for young G1 cells and its power in synchronizing cells from this stage onward, but also show that cell sorting, as a method for cell synchronization, poses little or no risk to the cell cycle or cell growth, at least for the cells used. These properties may be well appreciated by those who study the cell cycle, certainly in the context of cell size and growth.

Although we based our method on the sorting technology that has been available for decades, this study emerged from our recent observation that FSC-W or SSC-A, but not FSC-A, are preferable light scatter parameters for approximating cell size, at least in a uniform cell culture [10]. FSC-A, in contrast, lacks the accuracy required for effectively separating cells by size, certainly not within the ∼five-fold volume range existing in a population of proliferating cells of a certain type [2], [10].

**Figure 5:**
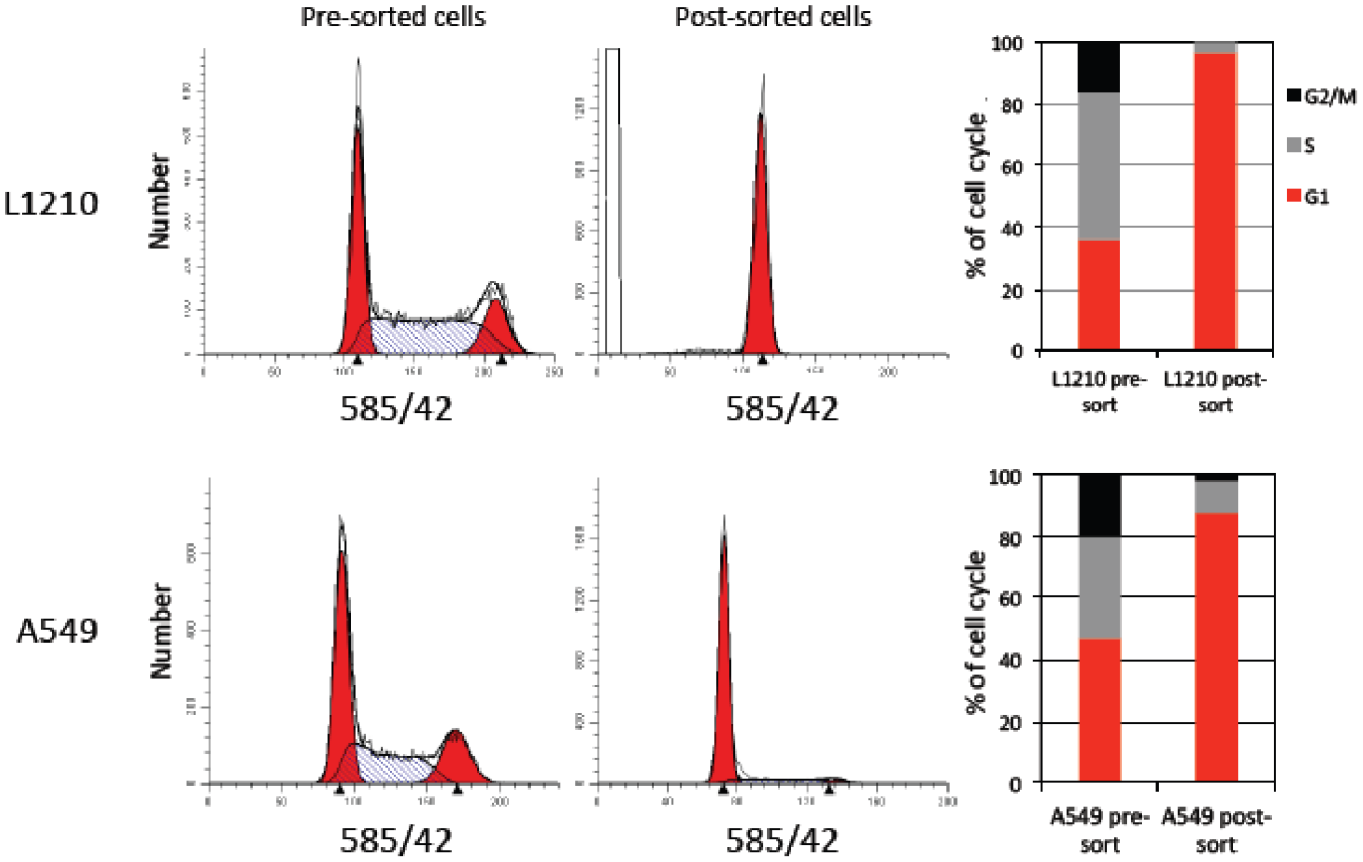
Size-based sorting of L1210 and A549 cells yields G1 cell populations. L1210 exhibiting the lowest 2.5% of the FSC-W distribution were gated for sorting (FACSAria III). Similarly, A549 cells exhibiting the lowest 10% of the SSC-A distribution were gated for sorting. Post-sorted and pre-sorted (unsynchronized) L1210 and A549 cells were then fixed and stained with PI for quantification of DNA and cell cycle progression (ModFit LT), which is also shown.

By relying on the natural optical scattering of the cell, our approach is independent of external, possibly toxic, reagents or drugs, and seems to pose minimal interference to both cell cycle and cell growth, as evident by the cell viability, cell growth, and cell cycle progression of the sorted cells (Figures 3 and 4). The level of synchronization can be appreciated post-sorting by the remarkable selectivity for G1 cells, as well as many hours after, as evident by the high fraction of cells in the S phase (>85%) and the significant enrichment of G2/M cells (∼50%) 17 and 26 hours post-sorting, respectively. The use of size-based sorting for G1 synchronization can be clearly extended to other adherent and unattached cell lines (Figure 5; A549 and L1210 cells, respectively). In fact, by sorting L1210 cells exhibiting the lowest 2.5% FSC-W [10], we can obtain G1-phase selectivity of 95–99%. This extreme selectivity is particularly impressive considering the short G1 phase that the L1210 cells normally have [2].

We minimized the sorting rate in order to maximize cell viability. Nevertheless, we were able to sort millions of HEK293 cells within half an hour without compromising on purity, cell health or the level of synchronization. Such a population size can be limiting for some biochemical applications, yet sufficient for a wide range of physiological, molecular and genomic studies. Lowering the percent of selected HEK293 cells below 8% of the FSC-W distribution was found to be insignificant with respect to both size and G1 selectivity, however it reduced the sorting yield proportionally (data not shown). This observation can be explained by the imperfect light scatter-to-size correlation and overall precision limit in asymmetric and complex particles, such as HEK293 cells. It may very well be that HEK293 and other cell types of a certain size, shape, and texture can tolerate higher sorting speed without compromising on cell viability or selectivity. By the same token, other cell types, in particular large adherent cells, may not tolerate even mildest sorting conditions. Naturally, such cells are incompatible with our method of synchronization. At this juncture, we also note that sorters without a flow cell (*e.g.,* MoFlo XPD/Astrios), are inherently optimized for high-speed sorting. Hence, sorting conditions, much like the light parameter(s) used to best approximate cell size [10], should be optimized empirically for each cell type and sorting instrument.

In relying on cell size, our approach would obviously benefit if cells were selected for their actual volume or mass rather than the light-scatter approximation of their size. This is particularly important for cells whose size correlates poorly with light scattering and currently cannot be properly synchronized by our approach. Technologies to directly measure cell volume (Coulter principle) or dry mass (interferometry) in living cells have been known for decades. The former was even implemented in commercial cytometers (*e.g.*, iCyt EC800). Yet, neither cell volume nor any other direct measure of cell size is available in conventional cell sorters. Interestingly, in the early days of optical flow cytometry, custom-designed sorters could separate cells by the Coulter principle. Perhaps the noticeable growing interest in cell size and its biological role in both proliferating and resting cells might revive the commercial production of such devices for biological applications and beyond.

## Materials and Methods

### Tissue Culture and Cell Synchronization

HEK293 cells (ATCC) were maintained in DMEM (Gibco) supplemented with 10% fetal bovine serum (Gibco) and 1% penicillin and streptomycin (Gibco) at 37°C, 5% CO_2_. Thymidine/nocodazole-induced synchronization was performed as follows: HEK293 cells at 30% confluence were treated with 2 mM thymidine (Sigma-Aldrich) for 20 hrs, washed twice with PBS, released for 3 hrs, and incubated with 100 ng/ml nocodazole (Sigma-Aldrich) for 12 hrs to generate a synchronous pre-metaphase cell population. Nocodazole release involved two washes (PBS) of the arrested cells and replating in fresh warm media, followed by harvesting at designated time points. The prometaphase cell extracts in Figure 3E were achieved by 15 hrs incubation with 100 ng/ml nocodazole.

### Cell Sorting, DNA Analysis, and Cell-volume Measurements

We used FACSAria III (BD) for cell sorting. In order to maximize cell viability and minimize mechanical perturbations, we set the flow rate to 1 (minimum), the pressure to 20 psi (minimum), and used an 85 µm nozzle with purity ranging between 75–90%.

For DNA staining in live cells, we incubated HEK293 cells with 1.8 mM Hoechst 33342 (MP Biomedicals) at 37°C for 30 min and used the FACSAria III (BD) 405 nm excitation laser for quantification. We followed a standard PI (Sigma-Aldrich) staining protocol for quantifying DNA in fixed HEK293 cells. We used the Gallios flow cytometer (Beckman Coulter) and 488 nm excitation laser for these measurements. Distribution of the cell cycle phases was determined using ModFit LT™ software for DNA and cell cycle analysis. We used a Multisizer™ IV Coulter counter with a 100 µm aperture (Beckman Coulter) for all cell volume measurements.

### Western Blotting

HEK293 cells were lysed using RIPA buffer (150 mM NaCl, 1.0% NP40, 0.5% sodium deoxycholate, 0.1% SDS, 50 mM Tris, pH 8.0) and processed for immunoblotting following a standard protocol. We used anti-Geminin (SC13015, Santa Cruz Biotechnology, Inc.), anti-Cdc20 (SC-8358, Santa Cruz Biotechnology, Inc.), anti-Actin (DSHB, JLA20) primary antibodies, and matched HRP-coupled secondary antibodies (Jackson ImmunoResearch).

## Supporting Information

Figure S1 **DNA distribution of synchronous HEK293 cells.** HEK293 cells exhibiting the lowest 8% FSC-W intensity were sorted (FACSAria III) and incubated in fresh, warm media for up to 44 hrs. Cells were harvested at the indicated time points, fixed, and stained with PI for quantifying DNA (Gallios). Raw data modeled by the Sync Wizard (ModFit LT) algorithm are depicted (ModFit LT). DNA quantification of pre-sorted, unsynchronized HEK293 cells (unsync) is also shown.
(DOCX)
