# Supplementary material for: Using Standard Optical Flow Cytometry for Synchronizing Proliferating Cells in the G1 Phase
Source: PLoS One. 2013 Dec 31;8(12):e83935. doi: 10.1371/journal.pone.0083935 (PMC3877101; doi:10.1371/journal.pone.0083935)
Supplement: Figure S1 — DNA distribution of synchronous HEK293 cells. HEK293 cells exhibiting the lowest 8% FSC-W intensity were sorted (FACSAria III) and incubated in fresh, warm media for up to 44 hrs. Cells were harvested at the indicated time points, fixed, and stained with PI for quantifying DNA (Gallios). Raw data modeled by the Sync Wizard (ModFit LT) algorithm are depicted (ModFit LT). DNA quantification of pre-sorted, unsynchronized HEK293 cells (unsync) is also shown. (DOCX) [file pone.0083935.s001.docx]

**Supplementary material for:**

***Using standard optical flow cytometry for***

***synchronizing proliferating cells in the G1 phase***

Manuela Vecsler^1,2^; Itay Lazar^1^; Amit Tzur^1,2,*^

**Figure S1**

**Figure S1:** *DNA distribution of synchronous HEK293 cells.* HEK293 cells exhibiting the lowest 8% FSC-W intensity were sorted (FACSAria III) and incubated in fresh, warm media for up to 44 hrs. Cells were harvested at the indicated time points, fixed, and stained with PI for quantifying DNA (Gallios). Raw data modeled by the Sync Wizard (ModFit LT) algorithm are depicted (ModFit LT). DNA quantification of pre-sorted, unsynchronized HEK293 cells (unsync) is also shown.
